# Accuracy and clinical impact of MRI in early-stage cervical cancer after cervical conization, a retrospective study

**DOI:** 10.1007/s00330-025-12178-9

**Published:** 2025-12-18

**Authors:** M. Dolciami, M. Criscione, N. Bizzarri, A. Amerighi, A. Napoletano, L. Russo, G. Scaglione, D. Giannarelli, M. Petrillo, E. Sala, F. Fanfani, B. Gui

**Affiliations:** 1 Department of Diagnostic Imaging and Radiation Oncology, Fondazione Policlinico Universitario Agostino Gemelli IRCCS, Rome, Italy; 2 Catholic University of the Sacred Heart, Rome, Italy; 3 Gynecologic and Obstetric Clinic, Department of Medicine, Surgery and Pharmacy, University of Sassari, Sassari, Italy; 4 Department of Women and Child Health and Public Health, UOC Gynecologic Oncology, Fondazione Policlinico Universitario Agostino Gemelli IRCCS, Rome, Italy; 5 Pathology Institute, Catholic University of the Sacred Heart, Rome, Italy; 6 Facility of Epidemiology and Biostatistics, G-SteP, Fondazione Policlinico Universitario Agostino Gemelli IRCCS, Rome, Italy

**Keywords:** Magnetic resonance imaging, Cervical cancer, Conization

## Abstract

**Objective:**

To assess the accuracy of MRI in detecting residual disease after conization in patients with early-stage cervical cancer (CC) and to evaluate the impact of MRI on FIGO staging and surgical management.

**Materials and methods:**

Between January 2018 and December 2023, a consecutive series of patients with early-stage invasive CC undergoing conization, pre-operative pelvic MRI, and surgery were included in this retrospective study. Two experienced radiologists reviewed the MRI scans for the presence of residual tumor, assessed on T2-weighted, diffusion-weighted, and contrast-enhanced sequences, if available. MRI findings were compared with surgical pathology to evaluate diagnostic performance and clinical impact.

**Results:**

A total of 108 patients were included in the study. MRI detected residual disease with an accuracy of 78.7% (95% CI 71.0–86.4), sensitivity of 66%, specificity of 90.9%, positive predictive value of 87.5%, and negative predictive value of 73.5%. The use of contrast agent showed no significant difference in overall accuracy, with an accuracy of 74.5% for contrast-enhanced MRI (CE-MRI) and 83% for non-CE-MRI (*p* = 0.28), respectively. MRI correctly staged 73.8% of cases, suggested the correct extent of radicality in 79.7% of cases following the 2018 ESGO guidelines, and the appropriate type of hysterectomy (simple vs. radical) in 90.9% of cases following the SHAPE study.

**Conclusion:**

Non-CE MRI is a reliable and sufficient tool for detecting residual tumor after conization in early-stage CC, as contrast administration offers no additional diagnostic value. Accurate pre-operative identification of residual disease may refine FIGO staging and assist in tailoring surgical strategies.

**Key Points:**

***Question*** *MRI evaluation of residual tumor after cervical conization in early-stage cervical cancer is critical to optimize surgical planning noninvasively and avoid overtreatment.*

***Findings*** *MRI correctly identified residual disease with high specificity (90.9%) and moderate sensitivity (66%); contrast enhancement did not significantly improve diagnostic accuracy.*

***Clinical relevance*** *MRI, particularly non-contrast protocols, reliably excludes post-conization residual disease and aids in selecting appropriate surgical treatment, reducing overtreatment in early-stage cervical cancer.*

**Graphical Abstract:**

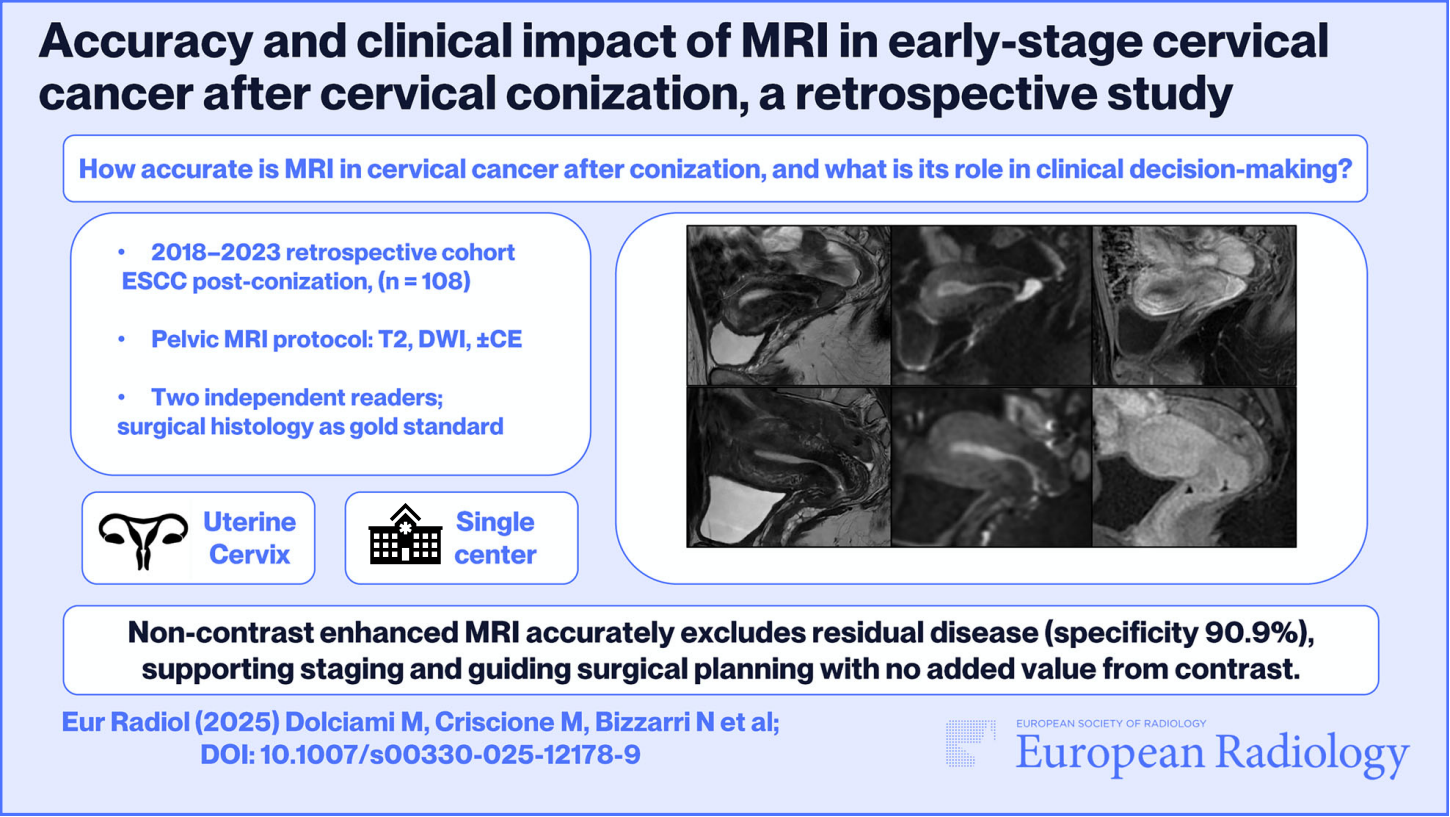

## Introduction

Cervical cancer (CC) remains a significant public health issue despite a notable decrease in incidence in developed countries due to the adoption of screening and vaccination programs [1, 2]. Over recent decades, prevention and early detection of precancerous lesions have led to a reduction in the incidence, mortality, and Disability-Adjusted Life Years (DALYs) due to CC globally, regionally, and nationally, increasing early diagnoses of small-volume disease [3, 4]. Standard clinical management of patients with abnormal cytology or positive human papillomavirus (HPV) testing typically includes colposcopy, followed by incisional or excisional procedures, such as loop electrosurgical excision (LEEP) or cold knife conization (CKC) [5, 6]. These diagnostic and therapeutic procedures aim for complete lesion excision, but incomplete resection may leave residual disease [7]. Accurate assessment of residual tumor post-conization is crucial for treatment planning, especially in fertility-sparing cases [8]. In 62–85% of early-stage CC patients undergoing trachelectomy after conization, no residual tumor is found [5, 9]. According to the International Federation of Gynecology and Obstetrics (FIGO) 2018, stage IA1-IB1 patients have a low risk of parametrial invasion and lymph node metastasis, making them suitable for less radical procedures [10–12].

Repeated invasive procedures, like conization or endocervical curettage, can confirm residual tumors but may negatively impact fertility, necessitating accurate non-invasive imaging. The 2018 FIGO staging system update introduces cross-sectional imaging for staging primary tumors, emphasizing Magnetic Resonance Imaging (MRI) for local extension assessment [2]. Due to its excellent soft tissue contrast, MRI has emerged as a key tool in evaluating primary tumor size, stromal infiltration, and invasion of parametria and adjacent organs [13, 14]. According to the 2023 guidelines from the European Society of Gynaecological Oncology (ESGO), the European Society for Radiotherapy and Oncology (ESTRO), and the European Society of Pathology (ESP), MRI is mandatory for staging and guiding treatment decisions in most stages CC (optional only in the case of T1a with clear margins after conization) [15]. The NCCN 2025 guidelines also suggest using MRI in the initial workup of CC for both stage I—whether or not patients are candidates for fertility-sparing treatment—and more advanced stages (II–IVA) [16].

In 2021, the European Society of Urogenital Radiology (ESUR) published updated guidelines for using MRI in CC, outlining the primary clinical indications, optimal patient preparation, technical requirements, and the recommended MRI protocol [17]. However, the role of MRI in staging after conization has not been clearly established, and only a few studies have addressed this topic, showing that integrating MRI findings with pathologic results from conization may increase diagnostic confidence in excluding residual disease and potentially reduce under- and overtreatment in patients with early-stage CC [5, 18–20]. After conization, MRI, including diffusion-weighted imaging (DWI), showed promising results for detecting residual disease due to its ability to differentiate tumors from post-conization findings, such as hemorrhage, edema, and inflammation [21–23]. This study aimed to evaluate the accuracy of MRI in detecting residual disease after conization in patients with clinically early-stage CC and the added value of contrast agent. Additionally, we aimed to assess the impact of MRI evaluation on staging and selecting the most appropriate surgical treatment.

## Materials and methods

### Study design and patient population

This is a retrospective, single-center study. The Institutional Ethics Committee at Fondazione Policlinico Universitario A. Gemelli IRCCS approved this study (no. 0023759/24 ID:7073).

A consecutive series of patients who underwent conization and subsequent surgical treatment between January 2018 and December 2023 was included

The inclusion criteria were: (1) > 18-year-old women; (2) clinically early-stage CC (FIGO 2018 IA1-IIA1, except IB3); (3) histologically proven CC at conization; (4) availability of post-conization MR imaging performed within 6 months before surgery as index test; (5) availability of histopathological result as reference standard after radical hysterectomy, trachelectomy, or re-conization. Exclusion criteria included: conization specimens showing pre-malignant lesions without invasive carcinoma; re-conization or trachelectomy specimens showing invasive carcinoma with positive margins; patients who previously received radiotherapy and/or chemotherapy for CC; patients with lymph node or distant metastasis; and patients with MRI scans deemed incomplete. The study and manuscript meet the STARD guidelines [24].

### Conization procedures, surgical treatment, and histopathologic results

Conization procedures were performed at both our and collaborating centers using CKC or LEEP. Data from cone biopsies included histology (squamous cell, adenocarcinoma, adenosquamous, other), grade (well, moderate, poor), margins, infiltration, tumor extent (mm), and FIGO stage (2018) when available. Post-surgery, all specimens were analyzed by a single gynecologic pathologist dedicated to this study, ensuring consistent and expert assessment of residual tumor presence, histology, grade, location, and extent. Tumor size was defined as the maximum diameter measured along the surface epithelium perpendicular to the stromal infiltration, while tumor invasion depth was measured perpendicular to the basement membrane of the surface epithelium; if the depth of stromal invasion was ≤ 5 mm, the lesion was also defined as minimally invasive. For multifocal disease, the dimension of the largest focus was recorded. When a residual tumor was present in the hysterectomy specimen, the invasion depth from the corresponding site in the conization specimen was summed to provide the best estimate. Staging was performed according to FIGO 2018 and the 9th edition American Joint Committee on Cancer (AJCC) system.

### MRI protocol and image analysis

Our Picture Archiving and Communication System (PACS) was searched to retrieve MRI exams of all patients who had previously received conization; MRI exams from other institutions previously reviewed by our multidisciplinary team for treatment planning were also included.

According to the 2021 European Society of Urogenital Radiology (ESUR) guidelines [17], to be eligible for inclusion, the MR protocol had to contain at least the following requirements: (1) minimum magnetic field strength of 1.5 Tesla (T); (2) at least two T2-weighted imaging (T2WI) sequences of the pelvis in the sagittal plane and para-axial plane (perpendicular to the cervical canal), with a maximum slice thickness of 4 mm; (3) a diffusion-weighted imaging (DWI) sequence in axial/para-axial plane, using at least 2 b values (low *b* = 0–50 s/mm^2^, high *b* = 800–1000 s/mm^2^). Post-contrast imaging was considered optional and noted when present.

**Fig. 1:**
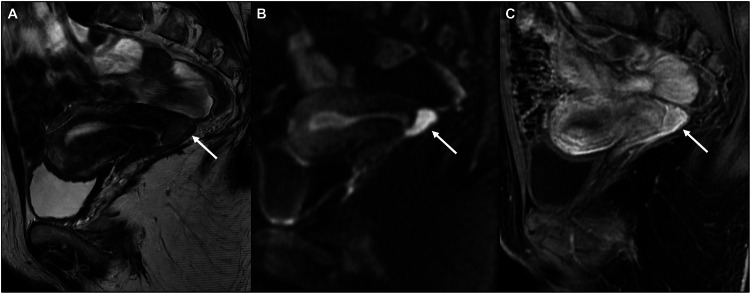
MR-visible residual tumor. Sagittal T2WI (**A**) shows a mass (white arrow) in the posterior cervical lip with intermediate signal intensity. The lesion shows restricted diffusion in DWI (**B**, white arrow) and marked contrast enhancement in post-contrast fat-saturated T1WI (**C**, white arrow). Final histology after hysterectomy confirmed squamous cell tumor

**Fig. 2:**
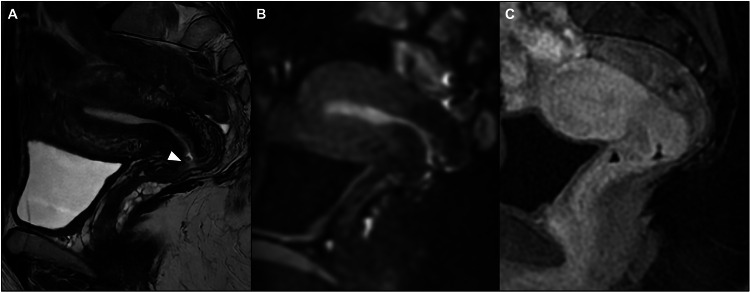
MR-invisible residual tumor. Sagittal T2WI (**A**) shows a small defect (white arrowhead) in the anterior cervical lip as a sign of a previous cone biopsy and no suspected cervical mass or nodularity. There are no restricted diffusion or abnormal contrast enhancement areas on DWI (**B**) and post-contrast fat-saturated T1WI (**C**), respectively. Final histology after trachelectomy confirmed no residual tumor

All images were assessed by two dedicated radiologists in consensus (B.G. and M.D., with 25 and 7 years of experience in gynecological imaging, respectively). The radiologists knew the patients had CC and had undergone conization before the MRI, but they were blinded to the clinical and pathological specifics. First, each MRI was evaluated for completeness of the protocol according to ESUR guidelines and excluded if deemed incomplete based on the criteria listed above. Then, the two radiologists assessed whether there was an identifiable residual tumor based on the combined evaluation of MR signal intensity on both T2WI and DWI, and if present, its dimensions. Images were deemed positive for residual tumor in the presence of a cervical mass or nodularity with intermediate/hyperintense signal in T2WI, corresponding to diffusion restriction in DWI and apparent diffusion coefficient (ADC) map (MR-visible) (Fig. 1). Images were considered negative for residual tumor if there was no mass or in case of nodularity in T2WI without corresponding diffusion restriction in DWI/ADC (MR-invisible) (Fig. 2). If contrast agent was administered, post-contrast signal characteristics were also evaluated on T1WI (hypointense, isointense, and hyperintense compared to the normal cervical stroma). Where residual tumor was visible on MR, the dimension was assessed on the sagittal and para-axial planes, reporting the maximum diameter on any plane. Furthermore, the presence/absence of parametrial invasion, adjacent organ infiltration, and suspicious lymph node metastases on MRI was assessed to exclude the erroneous inclusion of higher-stage patients.

### MRI prediction of staging and type of surgery

Patient staging was estimated by summing the tumor size from the conization specimen and the MRI maximum residual tumor dimension, when complete conization data were available. A descriptive analysis of the prediction (correctly staged, overstaged, or understaged) was then performed, comparing it to the final staging (combined data of conization and final surgical specimen). Patients were considered correctly staged, overstaged, or understaged if the stage determined by cone biopsy and MRI data corresponded to, was higher than, or was lower than the final post-surgical stage, respectively.

The recommended extent of radical hysterectomy, based on the sum of tumor size at conization plus MRI data, defined according to Querleu-Morrow, was estimated using the 2018 ESGO guidelines [25]. The ESGO criteria (tumor size, LVSI, stromal invasion) categorize patients into low, intermediate, and high-risk groups, with each group receiving a specific recommended type of surgery. The SHAPE study demonstrates that simple hysterectomy (SH) is not inferior to radical hysterectomy (RH) for the risk of pelvic recurrence but is superior for quality of life and sexual health, with economic savings. Applying the SHAPE criteria, we compared the predictive ability of MRI in providing the appropriate indication for performing SH. SHAPE trial included patients with squamous cell carcinoma, adenocarcinoma, or adenosquamous carcinoma of any grade, classified as FIGO 2009 stage IA2 or IB1. Eligible tumors measured ≤ 2 cm with stromal invasion < 10 mm on LEEP or conization, or < 50% stromal invasion on MRI. Nodal involvement was an exclusion criterion [26]. A descriptive prediction analysis (concordant treatment, overtreatment, or undertreatment) was then performed, comparing data from conization and MRI with the gold standard (combined data of conization and surgical specimen) for both the extent of radical hysterectomy according to ESGO guidelines and the selection of simple hysterectomy based on SHAPE criteria. The treatment choice, based on combined MRI and conization data, was classified as “concordant treatment,” “overtreatment,” or “undertreatment” depending on whether it matched, exceeded, or was less than the treatment decision derived from applying the combined data of conization and surgical specimen to ESGO guidelines and SHAPE criteria.

### Statistical analysis

Patients were divided into two groups (the MR-invisible group and the MR-visible group).

2 × 2 contingency tables (reflecting the number of TP = true positives, TN = true negatives, FP = false positives, and FN = false negatives) were extracted based on the final surgical specimen as the gold standard. Sensitivity (Se), Specificity (Sp), positive and negative predictive values (PPV, NPV), and accuracy of MRI in distinguishing residual tumor after cone biopsy/LEETZ were calculated. A further contingency table was created considering the minimally invasive tumor (depth of stromal infiltration ≤ 5 mm) as negative at the final surgical specimen. The Chi-square test was used to compare the accuracy between subgroups. A significance level of *p* < 0.05 was used to determine statistical significance. All statistical analyses were performed using SPSS software (version 29.0; SPSS Inc.).

## Results

### Patient population

**Fig. 3:**
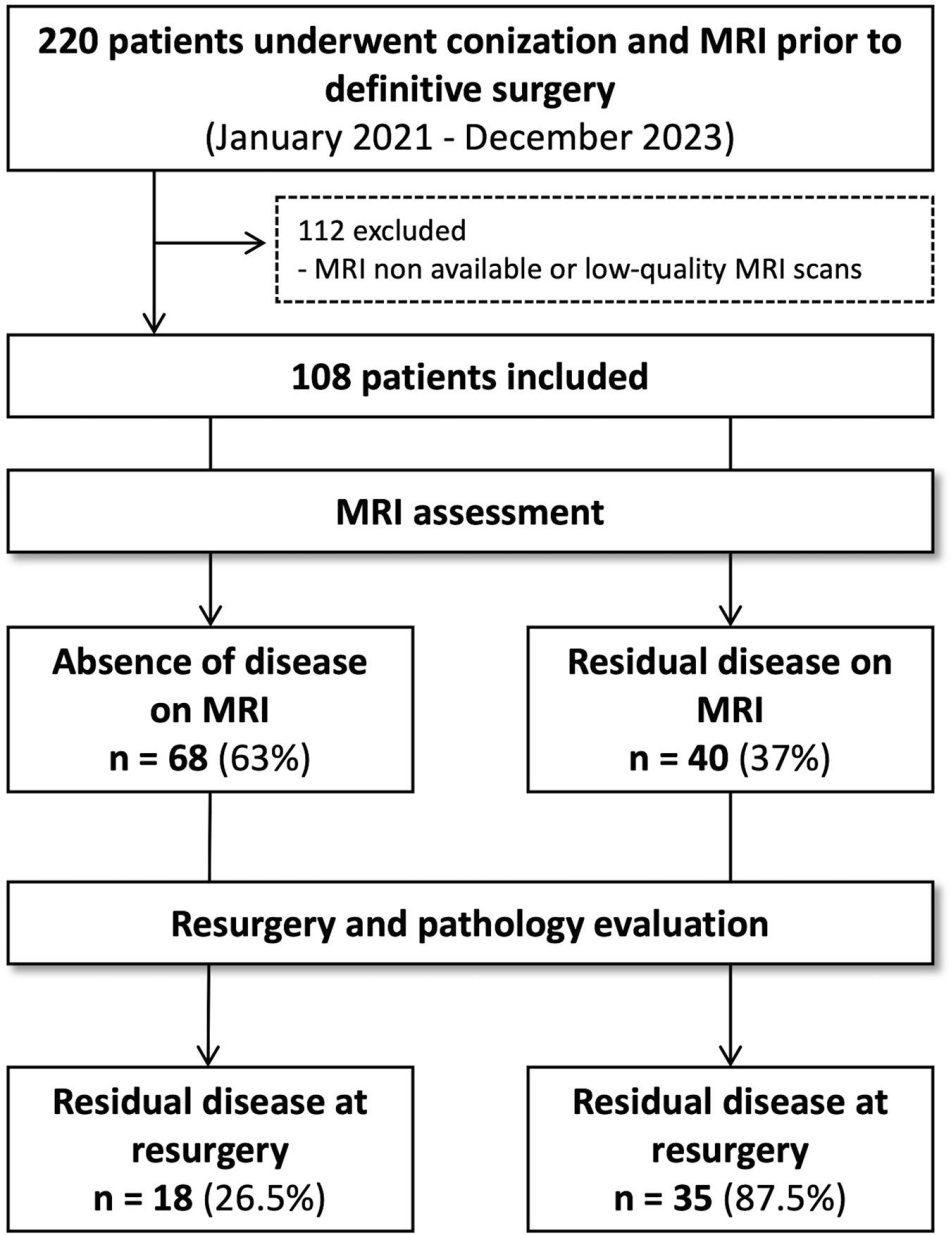
Flowchart for patient selection

The flowchart of patient selection is provided in Fig. 3.

A total of 108 patients with a median age of 44 years (18–86) were included (Table 1). Median time between conization and MRI scan was 43 days (IQR: 32–53), and surgery was performed with a median interval of 26.5 days after MRI (IQR: 7–41).

**Table 1:** Study population—clinical and pathological findings

Characteristics	Value (%)	
Total 108 patients	Non-visible disease on MRI *n* = 68 (63)	Visible disease on MRI *n* = 40 (37)
Median age at conization		
Median (IQR)	43.4 (38.5–48.9)	46.7 (39.6–51.7)
Average (range)	44.57 (14.9–67.9)	48 (28.5–86.1)
Margins at conization		
Positive	40 (58.8)	27 (67.5)
Negative	22 (32.4)	2 (5.0)
Not available	6 (8.82)	11 (27.5)
Pathologic types at conization		
Squamous cell carcinoma	44 (64.7)	27 (67.5)
Adenocarcinoma	20 (29.4)	10 (25.0)
Adenosquamous carcinoma	2 (2.9)	2 (5.0)
Not specified carcinoma	2 (2.9)	1 (2.5)
Type of definitive surgery		
Trachelectomy	1 (1.5)	1 (2.5)
Re-conization	9 (13.2)	2 (5.0)
Radical Hysterectomy	58 (85.3)	37 (92.5)
Hysterectomy type Q-M		
A	28 (48.3)	6 (16.2)
B1	8 (13.8)	2 (5.4)
B2	12 (20.7)	7 (18.9)
C1	10 (17.2)	22 (59.5)
Residual of disease at final surgery		
No	50 (73.5)	5 (12.5)
Yes	18 (26.5)	35 (87.5)
Residual tumor d-max at final surgery (mm)		
≤ 5	5 (27.8)	3 (8.6)
6–10	6 (33.3)	4 (11.4)
11–20	1 (5.5)	16 (45.7)
> 20	2 (11.1)	7 (20.0)
Not available	4 (22.2)	5 (14.3)
Residual tumor D.max mm median (IQR)	7.0 (4.3–9.8)	17.5 (12.3–20.0)
Residual tumor D.max mm average (range)	8.6 (2–24)	17.6 (1.5–46.0)
Residual tumor D.max mm median (IQR) tot	14.0 (7.8–20.0)	
Residual tumor D.max mm average (range) tot	14.76 (1.5–46.0)	
Stromal infiltration at final surgery		
Minimally invasive	14 (77.8)	15 (42.9)
Invasive	4 (22.2)	20 (57.1)
FIGO 2018 final staging		
IA1	27 (39.7)	5 (12.5)
IA2	18 (26.5)	5 (12.5)
IB1	15 (22.1)	12 (30.0)
IB2	7 (10.3)	12 (30.0)
IB3	0 (0.0)	1 (2.5)
IIA1	1 (1.5)	3 (7.5)
IIA2	0 (0.0)	1 (2.5)
IIB	0 (0.0)	1 (2.5)
LVSI		
Positive	15 (22.1)	19 (47.5)
Negative	28 (41.2)	12 (30.0)
Unknown	25 (36.8)	9 (22.5)

### Pathology of conization specimens

Minimally invasive carcinoma (≤ 5 mm stromal invasion) was found in 68/108 conization specimens (63%), and invasive carcinoma (≥ 5 mm) in 40 (37%). Positive margins were present in 67 cases (62%), and an unknown margin status was reported in 17 cases (15.7%). Histology revealed squamous cell carcinoma in 71 cases (65%), adenocarcinoma in 30 (27.8%), and adenosquamous carcinoma in 4 (3.7%). LVSI was positive in 34 cases (31.5%), negative in 40 (37%), and unknown in 34 (31.5%) (Table 1).

### Surgical procedure

Radical hysterectomy was the most common surgical procedure, with 95 cases (88%). Only 11 (10.2%) patients had repeat conization, and two (1.8%) underwent vaginal trachelectomy (Table 1).

### Pathology of final surgical specimens

Residual disease was absent in 55 patients (50.9%). Among those with residual tumors (53 patients), maximum diameter (D-max) was ≤ 5 mm in 8 cases (15.1%), 6–10 mm in 10 cases (18.9%), 11–20 mm in 17 cases (32.1%), and > 20 mm in 9 cases (17.0%). Tumor size was unavailable in 9 patients (17.0%).

The median D-max of residual tumors was 14 mm (IQR: 7.8–20.0 mm; range: 1.5–46 mm). Final surgical specimens revealed minimally invasive carcinoma in 29 patients (54.7%) and invasive carcinoma in 24 (45.3%) (Table 1).

### MRI findings

Contrast-enhanced MRI (CE-MRI) was performed in 55 patients (50.9%), while 53 (49.1%) underwent non-contrast MRI. No residual tumor was identified in 68 cases (63.0%). Among the 40 patients (37%) with MR-visible residual tumors, 26 had lesions measuring 6–20 mm, 4 had tumors ≤ 5 mm, and 10 had lesions > 20 mm. The overall median D-max on MRI was 14 mm (range: 3–38 mm) (Table 2).

**Table 2:** MRI findings

Characteristics	Value
MDC	
No	53 (49.1)
Yes	55 (50.9)
Diffusion restriction	
No	70 (64.8)
Yes	38 (35.2)
Residual tumor d-max at MRI (mm)	
≤ 5	4 (10.0)
≤ 10	15 (37.5)
6–20	26 (65.0)
> 10	25 (62.5)
> 20	10 (25.0)
Residual tumor d-max at MRI mm median (IQR)	14.0 (8.75–20.25)
Residual tumor d-max at MRI mm average (range)	15.15 (3–38)

### MRI accuracy

MRI detected residual disease with an accuracy of 78.7% (95% confidence interval (CI) 70.98–86.43), Sensitivity of 66% (95% CI 53.29–78.79), Specificity of 90.9% (95% CI 83.31–98.51), PPV of 87.5% (95% CI 77.25–97.75), and NPV of 73.5% (95% CI 63.04–84.02). In the sub-analysis, where minimally invasive residual (depth of stromal infiltration < 5 mm) was deemed negative in the final surgical specimen, MRI sensitivity increased to 83.3% (95% CI 62.62–95.26), resulting in a lower FN rate of 16.7% (95% CI 1.76–31.58). However, the FP rate increased to 23.8% (95% CI 14.70–32.92), while overall accuracy minimally decreased to 77.8% (95% CI 68.76–85.21) (Table 3).

**Table 3:** MRI accuracy

	Residual tumor on the final surgery specimen						
	No residual tumor			Residual tumor (minimally invasive + invasive)			
Identifiable tumor on MRI							
No	50			18			
Yes	5			35			
	**Sensitivity (95% CI)**	**Specificity (95% CI)**	**Accuracy (95% CI)**	**Positive predictive value (95% CI)**	**Negative predictive value (95% CI)**	**False positive ratio (95% CI)**	**False negative ratio (95% CI)**
	66.0 (53.3–78.8)	90.9 (83.3–98.5)	78.7 (71.0–86.4)	87.5 (77.3–97.8)	73.5 (63.0–84.0)	9.1 (1.5–16.7)	34.0 (21.2–46.7)
	**No residual tumor + minimally invasive**			**Residual tumor (only invasive)**			
Identifiable tumor on MRI							
No	64			4			
Yes	20			20			
	**Sensitivity (95% CI)**	**Specificity (95% CI)**	**Accuracy (95% CI)**	**Positive predictive value (95% CI)**	**Negative predictive value (95% CI)**	**False positive ratio (95% CI)**	**False negative ratio (95% CI)**
	83.3 (62.6–95.3)	76.2 (65.7–84.8)	77.8 (68.8–85.2)	50.0 (39.6–60.4)	94.1 (86.7–97.5)	23.8 (14.7–32.9)	16.7 (1.8–31.6)

The use of contrast agents showed no significant difference in the overall accuracy, of 74.5% on CE-MRI and 83% on non-CE-MRI (*p* = 0.28). No differences in accuracy were observed between age groups and between squamous and other histotypes. Cone margin status (positive/negative) and cone biopsy infiltration did not significantly affect MRI accuracy (*p* = 0.12 and *p* = 0.80, respectively) (Table 4).

**Table 4:** MRI accuracy and time between conization and MRI

Time between conization and MRI (days)	Sensitivity	Specificity	Accuracy	VPP	VPN	FP ratio	FN ratio
≤ 30 days	86.7% (69.5–100)	77.8% (50.6–100)	83.3% (68.4–98.2)	86.7% (69.5–100)	77.8% (50.6–100)	22.2% (0–49.4)	13.3% (0–30.5)
31–45	58.8% (35.4–82.2)	95.0% (85.4–100)	78.4%(65.1–91.6)	90.9% (73.9–100)	73.1% (56.0–90.1)	5.0% (0–14.6)	41.2% (17.8–64.6)
46–60	46.2% (19.1–73.2)	80.0% (55.2–100)	60.9% (40.9–80.8)	75.0% (45.0–100)	53.3% (28.1–78.6)	20.0% (0–44.8)	53.8% (26.7–80.9)
> 60	75.0% (45.0–100)	100.0% (n.e.)	91.7% (80.6–100)	100.0% (n.e.)	88.9% (74.4–100)	0% (n.e.)	25.0% (0–55.0)
Median	*p* = 0.10	*p* = 0.50	*p* = 0.38	*p* = 0.91	*p* = 0.43	*p* = 0.50	*p* = 0.10
< 43 days	76.9% (60.7–93.1)	88.0% (75.3–100)	82.4% (71.9–92.8)	87.0% (73.2–100)	78.6% (63.4–93.8)	12.0% (0–24.7)	23.1% (6.9–39.3)
≥ 43 days	55.6% (36.8–74.3)	93.3% (84.4–100)	75.4% (64.3–86.6)	88.2% (72.9–100)	70.0% (55.8–84.2)	6.7% (0–15.6)	44.4% (25.7–63.2)

MRI performed ≤ 30 days after conization had an accuracy of 83.3% versus 91.7% > 60 days, although no significant differences were found (*p* = 0.38) (Table 5).

**Table 5:** MRI accuracy and patients characteristics

	No (%)	Sensitivity	Specificity	Accuracy (*p*-value)	VPP	NPP	FP ratio	FN ratio
CE-MRI				*p* = 0.28				
Yes	55 (49.1)	53.6 (35.1–72.0)	96.3 (89.2–100)	74.5 (63.0–86.1)	93.8 (81.9–100)	66.7 (51.9–81.5)	3.7 (0–10.8)	46.4 (28.0–64.9)
No	53 (50.9)	80.0 (64.3–95.7)	85.7 (72.7–98.7)	83.0 (72.9–93.1)	83.3 (68.4–98.2)	82.8 (69.0–96.5)	14.3 (1.3–27.2)	20.0 (4.3–35.7)
Age (years)				*p* = 0.98				
< 44	34 (31.5)	62.5 (43.1–81.9)	92.9 (83.3–100)	78.8 (67.7–89.9)	88.2 (72.9–100)	74.3 (59.8–88.8)	7.1 (0–16.7)	37.5 (18.1–56.9)
≥ 44	74 (68.5)	69.0 (52.1–85.8)	88.9 (77.0–100)	78.6 (67.8–89.3)	87.0 (73.2–100)	72.7 (57.5–87.9)	11.1 (0–23.0)	31.0 (14.2–47.9)
Histologic subtype				*p* = 0.42				
Squamous cell carcinoma	71 (65.7)	70.6 (55.3–85.9)	91.9 (83.1–100)	81.7 (72.7–90.7)	88.9 (77.0–100)	77.3 (64.9–89.7)	8.1 (0–16.9)	29.4 (14.1–44.7)
Others	37 (34.3)	57.9 (35.7–80.1)	88.9 (74.4–100)	73.0 (58.7–87.3)	84.6 (65.0–100)	66.7 (47.8–85.5)	11.1 (0–25.6)	42.1 (19.9–64.3)
Positive conization margin				*p* = 0.12				
Yes	67 (62.0)	52.0 (32.4–71.6)	93.0 (85.4–100)	77.9 (68.1–87.8)	81.3 (62.1–100)	76.9 (65.5–88.4)	7.0 (0–14.6)	48.0 (28.4–67.6)
No	24 (22.2)	78.6 (63.4–93.8)	83.3 (62.2–100)	80.0 (67.6–92.4)	91.7 (80.6–100)	62.5 (38.8–86.2)	16.7 (0–37.7)	21.4 (6.2–36.6)
Conization infiltration				*p* = 0.80				
Minimally invasive	68 (63.0)	52.0 (32.4–71.6)	93.0 (85.4–100)	77.9 (68.1–87.8)	81.3 (62.1–100)	76.9 (65.5–88.4)	7.0 (0–14.6)	48.0 (28.4–67.6)
Invasive	40 (37.0)	78.6 (63.4–93.8)	83.3 (62.2–100)	80.0 (67.6–92.4)	91.7 (80.6–100)	62.5 (38.8–86.2)	16.7 (0–37.7)	21.4 (6.2–36.6)

#### Impact of MRI on staging

Tumor staging estimation based on tumor size at conization and MRI in 107 patients resulted in 79 cases (73.8%) correctly staged, 11 (10.3%) overstaged, and 17 (15.9%) understaged; one patient was not assessable by cone + MRI due to incomplete information at conization. When minimally invasive tumors were considered negative, staging accuracy increased by only one case, reducing understaging to 16 patients (14.9%), with no change in overstaging (Supplementary Tables 1.1–1.3).

#### Impact of MRI on the extent of radicality

Based on the ESGO guidelines, 74 patients with known LVSI were selected. MRI results accurately assessed the extent of radicality in 59 (79.7%) patients. Six (8.1%) were at risk of overtreatment, and nine (12.2%) patients were at risk of undertreatment. Identical results were observed by considering minimally invasive cases as negative (Supplementary Tables 2.1, 2.2).

According to the SHAPE study criteria, 99 patients were selected. MRI findings facilitated the appropriate choice of hysterectomy type (simple vs. radical) for 90 (90.9%) patients. Three (3%) patients were at risk of overtreatment, while six patients (6.1%) were at risk of undertreatment. Almost identical results were noted when classifying minimally invasive cases as negative (Supplementary Tables 3.1, 3.2).

## Discussion

This study evaluated post-conization MRI’s role in early-stage CC, focusing on its accuracy in identifying residual tumors and influencing treatment choices.

MRI demonstrated an overall accuracy of 78.7% for detecting residual disease after conization, with moderate sensitivity (66%) and high specificity (90.9%). When minimally invasive cases were considered negative, sensitivity increased to 83.3%, and the FN ratio dropped to 16.7% (from 34.0%), highlighting the challenge of detecting very small tumors that can lead to false negatives. MRI performance was not significantly affected by contrast administration, nor by variables such as margin status or infiltration type in cone-biopsy specimens, patients’ age, or the interval between conization and MRI. Combined MRI and cone biopsy data correctly predicted FIGO stage (2018) in 73.2% of cases, with 15.7% understaged and 10.2% overstaged. Based on ESGO and SHAPE criteria, MRI correctly guided hysterectomy type in 79.7% and 90.9% of cases, respectively, with low overtreatment (8.1%, 3%) and undertreatment rates (12.2%, 6.1%).

Our results are consistent with previous studies and indicate that MRI, combining both T2WI and DWI sequences, is highly specific for ruling out residual disease. Woo et al found that MR-visible tumors independently predicted residual disease, with high specificity (92%) and low sensitivity (53%) [20]. Using an endovaginal coil, Charles-Edwards et al studied the accuracy of MRI in 85 patients with IA/IB CC, including both those who underwent conization and those who did not, finding an MRI sensitivity of 80% and specificity of 94.7% [21]. Similarly, a more recent study by Roh et al demonstrated a sensitivity of 63.5% and a specificity of 92% in CC with FIGO 2009 stage IA2-IB1 [23]. While the specificity is similar to our work, both studies showed higher sensitivity. However, we must note that the study by Charles-Edwards et al used an endovaginal coil instead of an external coil, and both studies involved smaller, mixed patient groups, including those who had undergone cone biopsy and those who had not, whereas all our patients had conization. Cervical conization causes post-surgical changes and inflammation, which can impair the MRI’s ability to detect residual tumors, thereby reducing our sensitivity.

These findings suggest that while post-conization MRI showed high specificity, its sensitivity is only moderate and carries a relatively high FN rate (34%). Review of the FN cases showed that the majority (14 out of 18; 78%) had stromal invasion ≤ 5 mm, a range that likely includes lesions whose subtle infiltration lies at or below the spatial resolution threshold of MRI. This limitation is further underscored by the results of a dedicated sub-analysis in which minimally invasive tumors were reclassified as negative: under this condition, sensitivity increased to 83.3%, and the false-negative rate decreased by 16.7%, while overall diagnostic accuracy minimally decreased to 77.8.

Our study showed that CE-MRI did not change diagnostic accuracy over non-contrast MRI (*p* = 0.28), with a nonsignificant increase in FN. We believe these findings may result from post-procedural inflammatory changes, appearing as increased enhancement in post-contrast images that extend into the cervical tissue, potentially obscuring the residual tumor. This aligns with previous studies suggesting DWI and T2-weighted sequences alone may suffice for CC assessment [27]. To our knowledge, this is the only study examining the qualitative CE-MRI’s value in predicting residual tumors after conization. Huang et al explored the utility of quantitative dynamic contrast-enhanced-MRI parameters, finding Ktrans and Ve were higher in patients with residual tumors [28]. However, quantitative DCE-MRI analysis is challenging and not widely available in clinical practice, making its results not comparable to our focus on qualitative evaluation. This suggests non-contrast MRI could be a preferred option for staging, follow-up, and post-conization CC, reducing unnecessary contrast administration and associated costs and risks (i.e., adverse/allergic reactions or nephrogenic systemic fibrosis, NSF).

Woo et al found that older patient age and positive margins at cone biopsy significantly correlated with residual tumors in final pathological specimens [20]. However, our work did not reveal any notable impact on MRI accuracy across various age groups, nor did it show significant differences in margin characteristics and infiltration in the cone biopsy specimens. We also attempted to evaluate whether differences in timing between conization and MRI influenced MRI performance, again without identifying significant results.

The evaluation of the clinical impact of MRI on staging, with 17 patients understaged and 11 overstaged, highlights the current need to consider other factors and diagnostic tools in the decision-making process, such as in the fertility-sparing treatment setting. The application of the ESGO criteria and the SHAPE study goes beyond proof of concept and concretely evaluates the clinical impact of MRI in determining the most suitable surgical approach for patients undergoing non-fertility-sparing surgery. Notably, MRI results according to the SHAPE study showed 90.9% (90 patients) agreement in the type of hysterectomy planned between the MRI-based and surgery-based groups, and 9.1% disagreement (6 patients overtreated with RH instead of SH, 3 patients undertreated with SH instead of RH). This approach overcomes the current diagnostic role, giving MRI a decisive role in establishing the extent of surgery, thus improving cost-effectiveness and quality of life [29]. Furthermore, the evaluation of MRI’s clinical impact on surgical decision-making, performed both in the overall population and after excluding minimally invasive cases, yielded identical and remarkable results (concordance of 79.7% in determining the extent of radicality and 90.9% in selecting the type of hysterectomy). This finding suggests that although MRI demonstrated a relatively high false-negative rate (34%) across the overall population, imaging-based stratification still led to the correct therapeutic choice in most patients. These results indicate that false-negative MRI findings, largely associated with minimally invasive disease, have limited influence on treatment allocation and patient safety, reinforcing MRI’s clinical value in guiding appropriate surgical management.

This study has several limitations. First, the retrospective single-center design may have introduced selection bias, as patient inclusion relied on the availability of MRI and histopathological data, potentially limiting our results, particularly in assessing the optimal timing between conization and MRI. Second, MRI examinations were performed using different scanners and imaging protocols, which may have led to variability in image quality and interpretation. Another limitation is the lack of interobserver agreement assessment. Although two experienced radiologists assessed MRI findings in consensus, interobserver agreement was not evaluated; future studies should include reproducibility analyses. Additionally, our subgroup analyses were underpowered for small-to-moderate differences. Specifically, detecting ~9% absolute difference in accuracy with 80% power (α = 0.05, two-sided) would require on the order of ~315 patients per group.

Last, although there are no definite guidelines, the impact of variable timing between MRI and surgical procedures has not been evaluated.

In conclusion, non-contrast MRI demonstrates high specificity and good accuracy in detecting residual disease after conization, supporting its role in guiding surgical decisions and reducing risks of over- or undertreatment. The lack of added value from CE-MRI suggests that non-contrast protocols may suffice for routine use. Prospective studies with larger cohorts and standardized protocols are needed to confirm these findings and refine MRI-based patient stratification, including optimal timing post-conization.

## Supplementary information

ELECTRONIC SUPPLEMENTARY MATERIAL

## Abbreviations

ADC: Apparent diffusion coefficient
CC: Cervical cancer
CE-MRI: Contrast-enhanced magnetic resonance imaging
D-max: Maximum diameter
DWI: Diffusion-weighted imaging
ESGO: European Society of Gynaecological Oncology
ESUR: European Society of Urogenital Radiology
FIGO: International Federation of Gynecology and Obstetrics
FN: False negative
FP: False positive
IQR: Interquartile range
LEEP: Loop electrosurgical excision procedure
LVSI: Lymphovascular space invasion
MRI: Magnetic resonance imaging
PPV: Positive predictive value
RH: Radical hysterectomy
SH: Simple hysterectomy
T: Tesla (unit of magnetic field strength)
T1W: T1-weighted imaging
T2WI: T2-weighted imaging

## References

[1] deSouza NM, Soutter WP, McIndoe GA, Gilderdale DJ, Krausz T (1997) Stage I cervical cancer: tumor volume by magnetic resonance imaging of screen-detected versus symptomatic lesions. J Natl Cancer Inst 89:1314–1315. 10.1093/jnci/89.17.1314 9293923 10.1093/jnci/89.17.1314

[2] Bhatla N, Denny L (2018) FIGO cancer report 2018. Int J Gynecol Obstet 143:2–3. 10.1002/ijgo.12608 10.1002/ijgo.12608 30306587

[3] Zhang X, Zeng Q, Cai W, Ruan W (2021) Trends of cervical cancer at global, regional, and national level: data from the Global Burden of Disease study 2019. BMC Public Health 21:894. 10.1186/s12889-021-10907-5 33975583 10.1186/s12889-021-10907-5 PMC8114503

[4] Bhatla N, Aoki D, Sharma DN, Sankaranarayanan R (2021) Cancer of the cervix uteri: 2021 update. Int J Gynaecol Obstet 155:28–44. 10.1002/ijgo.13865 34669203 10.1002/ijgo.13865 PMC9298213

[5] Lakhman Y, Akin O, Park KJ et al (2013) Stage IB1 cervical cancer: role of preoperative MR imaging in selection of patients for fertility-sparing radical trachelectomy. Radiology 269:149–158. 10.1148/radiol.13121746 23788721 10.1148/radiol.13121746 PMC6822769

[6] Schmeler KM, Frumovitz M, Ramirez PT (2011) Conservative management of early stage cervical cancer: is there a role for less radical surgery? Gynecol Oncol 120:321–325. 10.1016/j.ygyno.2010.12.352 21320670 10.1016/j.ygyno.2010.12.352 PMC4260451

[7] Petry KU (2011) Management options for cervical intraepithelial neoplasia. Best Pract Res Clin Obstet Gynaecol 25:641–651. 10.1016/j.bpobgyn.2011.04.007 21723198 10.1016/j.bpobgyn.2011.04.007

[8] van Hanegem N, Barroilhet LM, Nucci MR, Bernstein M, Feldman S (2012) Fertility-sparing treatment in younger women with adenocarcinoma in situ of the cervix. Gynecol Oncol 124:72–77. 10.1016/j.ygyno.2011.09.006 22030403 10.1016/j.ygyno.2011.09.006

[9] Plante M, Renaud M-C, Hoskins IA, Roy M (2005) Vaginal radical trachelectomy: a valuable fertility-preserving option in the management of early-stage cervical cancer. A series of 50 pregnancies and review of the literature. Gynecol Oncol 98:3–10. 10.1016/j.ygyno.2005.04.014 15936061 10.1016/j.ygyno.2005.04.014

[10] Xu M, Xie X, Cai L, Xie Y, Gao Q, Sun P (2022) Risk factor assessment of lymph node metastasis in patients with FIGO stage IB1 cervical cancer. Front Oncol 12:809159. 10.3389/fonc.2022.809159 35433446 10.3389/fonc.2022.809159 PMC9007329

[11] Baiocchi G, de Brot L, Faloppa CC et al (2017) Is parametrectomy always necessary in early-stage cervical cancer? Gynecol Oncol 146:16–19. 10.1016/j.ygyno.2017.03.514 28392128 10.1016/j.ygyno.2017.03.514

[12] Minig L, Fagotti A, Scambia G et al (2018) Incidence of lymph node metastases in women with low-risk early cervical cancer (<2 cm) without lymph-vascular invasion. Int J Gynecol Cancer 28:788–793. 10.1097/IGC.0000000000001236 29538254 10.1097/IGC.0000000000001236

[13] Salib MY, Russell JHB, Stewart VR et al (2020) 2018 FIGO staging classification for cervical cancer: added benefits of imaging. Radiographics 40:1807–1822. 10.1148/rg.2020200013 32946322 10.1148/rg.2020200013

[14] Russo L, Pasciuto T, Lupinelli M et al (2024) The value of MRI in quantification of parametrial invasion and association with prognosis in locally advanced cervical cancer: the “PLACE” study. Eur Radiol 34:4003–4013. 10.1007/s00330-023-10443-3 37981591 10.1007/s00330-023-10443-3

[15] Cibula D, Raspollini MR, Planchamp F et al (2023) ESGO/ESTRO/ESP guidelines for the management of patients with cervical cancer—update 2023. Virchows Arch 482:935–966. 10.1007/s00428-023-03552-3 37145263 10.1007/s00428-023-03552-3 PMC10247855

[16] National Comprehensive Cancer Network (NCCN) (2025) Cervical cancer, version 2.2025 https://www.nccn.org/professionals/physician_gls/pdf/cervical.pdf

[17] Manganaro L, Lakhman Y, Bharwani N et al (2021) Staging, recurrence and follow-up of uterine cervical cancer using MRI: updated guidelines of the European Society of Urogenital Radiology after revised FIGO staging 2018. Eur Radiol 31:7802–7816. 10.1007/s00330-020-07632-9 33852049 10.1007/s00330-020-07632-9

[18] Park J-Y, Lee J-W, Park BK et al (2014) Postoperative outcomes of MR-invisible stage IB1 cervical cancer. Am J Obstet Gynecol 211:168.e1–168.e7. 10.1016/j.ajog.2014.02.032 24607752 10.1016/j.ajog.2014.02.032

[19] Lee J-Y, Youm J, Kim J-W et al (2015) Identifying a low-risk group for parametrial involvement in microscopic stage IB1 cervical cancer using criteria from ongoing studies and a new MRI criterion. BMC Cancer 15:167. 10.1186/s12885-015-1184-2 25885786 10.1186/s12885-015-1184-2 PMC4374417

[20] Woo S, Kim HS, Chung HH, Kim SY, Kim SH, Cho JY (2016) Early stage cervical cancer: role of magnetic resonance imaging after conization in determining residual tumor. Acta Radiol 57:1268–1276. 10.1177/0284185115620948 26671305 10.1177/0284185115620948

[21] Charles-Edwards E, Morgan V, Attygalle AD et al (2011) Endovaginal magnetic resonance imaging of stage 1A/1B cervical cancer with A T2- and diffusion-weighted magnetic resonance technique: effect of lesion size and previous cone biopsy on tumor detectability. Gynecol Oncol 120:368–373. 10.1016/j.ygyno.2010.10.013 21093895 10.1016/j.ygyno.2010.10.013

[22] Li X, Wang L, Li Y, Song P (2017) The value of diffusion-weighted imaging in combination with conventional magnetic resonance imaging for improving tumor detection for early cervical carcinoma treated with fertility-sparing surgery. Int J Gynecol Cancer 27:1761–1768. 10.1097/IGC.0000000000001113 28930805 10.1097/IGC.0000000000001113

[23] Roh HJ, Go EB, Kim KBin, Lee JH, Lee SH (2019) The diagnostic accuracy and postoperative outcomes of cervical cancer patients for MR-invisible or MR-visible diagnosis of combined T2- And diffusion-weighted 3T MRI using the external phased-array receiver. Anticancer Res 39:6945–6956. 10.21873/anticanres.13916 31810966 10.21873/anticanres.13916

[24] Cohen JF, Korevaar DA, Altman DG et al (2016) STARD 2015 guidelines for reporting diagnostic accuracy studies: explanation and elaboration. BMJ Open 6:e012799. 10.1136/bmjopen-2016-012799 28137831 10.1136/bmjopen-2016-012799 PMC5128957

[25] Cibula D, Fischerova D, Potter R et al (2018) The European Society of Gynaecological Oncology/European Society for Radiotherapy and Oncology/European Society of Pathology guidelines for the management of patients with cervical cancer. Int J Gynecol Cancer 28:641–655. 10.1097/IGC.0000000000001216 29688967 10.1097/IGC.0000000000001216

[26] Plante M, Kwon JS, Ferguson S et al (2024) Simple versus radical hysterectomy in women with low-risk cervical cancer. N Engl J Med 390:819–829. 10.1056/NEJMoa2308900 38416430 10.1056/NEJMoa2308900

[27] Avesani G, Perazzolo A, Amerighi A et al (2023) The utility of contrast-enhanced magnetic resonance imaging in uterine cervical cancer: a systematic review. Life 13:1368. 10.3390/life13061368 37374150 10.3390/life13061368 PMC10303560

[28] Huang JW, Song JC, Chen T, Yang M, Ma ZL (2019) Making the invisible visible: improving detectability of MRI-invisible residual cervical cancer after conisation by DCE-MRI. Clin Radiol 74:166.e15–166.e21. 10.1016/j.crad.2018.10.013 30503642 10.1016/j.crad.2018.10.013

[29] Kwon JS, McTaggart-Cowan H, Ferguson SE et al (2024) Cost-effectiveness analysis of simple hysterectomy compared to radical hysterectomy for early cervical cancer: analysis from the GCIG/CCTG CX.5/SHAPE trial. J Gynecol Oncol 35:e117. 10.3802/jgo.2024.35.e117 39453395 10.3802/jgo.2024.35.e117 PMC11543249

