# Supplementary material for: Accuracy and clinical impact of MRI in early-stage cervical cancer after cervical conization, a retrospective study
Source: Eur Radiol. 2025 Dec 18;36(5):3442–53. doi: 10.1007/s00330-025-12178-9 (PMC13086706; doi:10.1007/s00330-025-12178-9)
Supplement: Supplementary file 1 — ELECTRONIC SUPPLEMENTARY MATERIAL [file 330_2025_12178_MOESM1_ESM.pdf]

Accuracy and clinical impact of MRI in early-stage cervical cancer after cervical conization, a retrospective study.

ELECTRONIC SUPPLEMENTARY MATERIAL

Eur Radiol (2025) Dociami M, Criscione M, Bizzarrri N, et al.

Supplementary Tables

**Supplementary Table 1.1.** Tumor staging estimation based on tumor size at conization and MRI vs tumor staging at resurgery		
Total no, of patients: 108	**No of patients (%)**	**Overstaged /Understaged/No difference**
**Invasive and minimally invasive** as positive	**11 (10.3)**	**OVERSTAGED**
	**17 (15.9)**	**UNDERSTAGED (ADVANCEMENT IN STAGING AT RESURGERY)**
	**79 (73.8)**	**NO DIFFERENCE IN STAGE**
**Invasive** as positive (minimally invasive as negative)	**11 (10.3)**	**OVERSTAGED**
	**16* (14.9)**	**UNDERSTAGED (ADVANCEMENT IN STAGING AT RESURGERY)**
	**80 (74.8)**	**NO DIFFERENCE IN STAGE**
1 patient without staging after conization		

**Supplementary Table 1.2.** Tumor staging estimation based on tumor size at conization and MRI vs tumor staging at resurgery, in true positives and true negatives. (details)				
Total no, of patients: 108		**No of patients (%)**	**Staging based on tumor size Conization + MRI**	**Staging based on tumor size Conization + resurgery**
**TRUE POSITIVE**	**True positive (35)** *considering invasive and minimally invasive as positive*	**8 (22.9)**	**OVERSTAGED**	
		3	IB1	IA1
		2	IB1	IA2
		3	IB2	IB1
		**7 (20.0)**	**UNDERSTAGED (ADVANCEMENT IN STAGING AT RESURGERY)**	
		1	IB1	IB2
		1	IB1	IIA1
		1	IB1	IB2
		2	IB2	IIA1
		1	IB2	IIA2
		1	IB2	IIB
		**20 (57.1)**	**NO DIFFERENCE IN STAGE**	
		1	IA1	IA1
		1	IA2	IA2
		7	IB1	IB1
		10	IB2	IB2
		1	IB3	IB3
	**True positive (20)** *considering only invasive (minimally invasive as negative)*	**3 (15.0)**	**OVERSTAGED**	
		1	IB1	IA2
		2	IB2	IB1
		**6 (30.0)**	**UNDERSTAGED (ADVANCEMENT IN STAGING AT RESURGERY)**	
		2	IB1	IB2
		2	IB2	IIA1
		1	IB2	IIA2
		1	IB2	IIB
		**11 (55.0)**	**NO DIFFERENCE IN STAGE**	
		3	IB1	IB1
		7	IB2	IB2
		1	IB3	IB3
**TRUE NEGATIVE**	**True negative (50)** *considering invasive and minimally invasive as positive*	**50 (100.0)**	**NO DIFFERENCE IN STAGE**	
		25	IA1	IA1
		14	IA2	IA2
		9	IB1	IB1
		2	IB2	IB2
	**True negative (64)** *considering only invasive (considering minimally invasive as negative)*	**5 (7.8)**	**UNDERSTAGED (ADVANCEMENT IN STAGING AT RESURGERY)**	
		1	IA1	IA2
		2	IA2	IB1
		2	IB1	IB2
		**58 (90.6)**	**NO DIFFERENCE IN STAGE**	
		27	IA1	IA1
		16	IA2	IA2
		11	IB1	IB1
		3	IB2	IB2
		**1 PATIENT WITHOUT STAGING AFTER CONIZATION**		

**Supplementary Table 1.3.** Tumor staging estimation based on tumor size at conization and MRI vs tumor staging at resurgery, in false positives and false negatives. (details)				
Total no, of patients: 108		**No of patients (%)**	**Staging based on tumor size Conization + MRI**	**Staging based on tumor size Conization + resurgery**
**FALSE POSITIVE**	**False positive (5)**<br>*considering invasive and minimally invasive as positive*	**3 (60.0)**	**OVERSTAGED**	
		2	IB1	IA1
		1	IB1	IA2
		**2 (40.0)**	**NO DIFFERENCE IN STAGE**	
		2	IB1	IB1
	**False positive (20)**<br>*considering only invasive*<br>*(minimally invasive as negative)*	**8 (40.0)**	**OVERSTAGED**	
		4	IB1	IA1
		3	IB1	IA2
		1	IB2	IB1
		**1 (5.0)**	**UNDERSTAGED (ADVANCEMENT IN STAGING AT RESURGERY)***	
		1	IB1	IIA1
		**11 (55.0)**	**NO DIFFERENCE IN STAGE**	
		1	IA1	IA1
		1	IA2	IA2
		6	IB1	IB1
		3	IB2	IB2
**FALSE NEGATIVE**	**False negative (18)**<br>*considering invasive and minimally invasive as positive*	**10 (55.6)**	**UNDERSTAGED (ADVANCEMENT IN STAGING AT RESURGERY)**	
		2	IA1	IA2
		2	IA1	IB1
		2	IA2	IB1
		1	IA2	IB2
		3	IB1	IB2
		**7 (38.9)**	**NO DIFFERENCE IN STAGE (NO ADVANCEMENT IN STAGING AT RESURGERY)**	
		2	IA1	IA1
		1	IA2	IA2
		2	IB1	IB1
		1	IB2	IB2
		1	IIA1	IIA1
		**1 PATIENT WITHOUT STAGING AFTER CONIZATION**		
	**False negative (4)**<br>*considering only invasive*<br>*(minimally invasive as negative)*	**4 (100.0)**	**UNDERSTAGED (ADVANCEMENT IN STAGING AT RESURGERY)**	
		1	IA1	IB1
		1	IA2	IB2
		2	IB1	IB2

*positivity, on resurgery specimen, of the posterior vaginal fornix

**Supplementary Table 2.1.** Radical hysterectomy indication (conization + MRI VS resurgery specimen) according ESGO 2018 guidelines		
Total no, of patients: 74	**No of patients (%)**	**overtreated / undertreated/ concordance**
**Invasive and minimally invasive** as positive	**6 (8.1)**	**OVERTREATED**
	**9 (12.2)**	**UNDERTREATED**
	**59 (79.7)**	**CONCORDANCE**
**Invasive** as positive (minimally invasive as negative)	**6 (8.1)**	**OVERTREATED**
	**9 (12.2)**	**UNDERTREATED**
	**59 (79.7)**	**CONCORDANCE**

**SupplementaryTable 2.2** Radical hysterectomy indication (conization + MRI VS resurgery specimen) according ESGO 2018 guidelines.					
Total no, of patients: 74		**No of patients**	**Type of RH based on tumor size Conization + MRI**	**Type of RH based on tumor size Conization + resurgery**	**No. of patients (%) - overtreated / undertreated/ Concordance**
**TRUE POSITIVE**	**True positive (30)** *considering invasive and minimally invasive as positive*	2	B2 (C1)	B1 (A)	6 (20.0) – Overtreated
		4	C1 (C2)	B2 (C1)	
		1	B1 (A)	B2 (C1)	3 (10.0) – Undertreated
		2	B2 (C1)	C1 (C2)	
		1	B1 (A)	B1 (A)	21 (70.0) - Concordance
		3	C1 (C2)	C1 (C2)	
		17	B2 (C1)	B2 (C1)	
	**True positive (18)** *considering only invasive as positive* *(minimally invasive as negative)*	3	C1 (C2)	B2 (C1)	3 (16.7) – Overtreated
		1	B1 (A)	B2 (C1)	3 (16.7) – Undertreated
		2	B2 (C1)	C1 (C2)	
		9	B2 (C1)	B2 (C1)	12 (66.7) - Concordance
		3	C1 (C2)	C1 (C2)	
**TRUE NEGATIVE**	**True negative (29)** *considering invasive and minimally invasive as positive*	21	B1 (A)	B1 (A)	29 (100.0)- Concordance
		6	B2 (C1)	B2 (C1)	
		2	C1 (C2)	C1 (C2)	
	**True negative (39)** *considering only invasive as positive (considering minimally invasive as negative)*	2	B1 (A)	B2 (C1)	3 (7.7) – Undertreated
		1	B2 (C1)	C1 (C2)	
		24	B1 (A)	B1 (A)	36 (92.3) - Concordance
		9	B2 (C1)	B2 (C1)	
		3	C1 (C2)	C1 (C2)	
**FALSE POSITIVE**	**False positive (1)** *considering invasive and minimally invasive as positive*	1	B2 (C1)	B2 (C1)	1 (100.0) - Concordance
	**False positive (13)** *considering only invasive as positive* *(minimally invasive as negative)*	2	B2 (C1)	B1 (A)	3 (23.1) – Overtreated
		1	C1 (C2)	B2 (C1)	
		1	B1 (A)	B1 (A)	10 (76.9) - Concordance
		9	B2 (C1)	B2 (C1)	
**FALSE NEGATIVE**	**False negative (14)** *considering invasive and minimally invasive as positive*	2	B1 (A)	B2 (C1)	6 (42.9) - Undertreated
		4	B2 (C1)	C1 (C2)	
		3	B1 (A)	B1 (A)	8 (57.1) - Concordance
		4	B2 (C1)	B2 (C1)	
		1	C1 (C2)	C1 (C2)	
	**False negative (4)** *considering only invasive as positive (minimally invasive as negative)*	1	B1 (A)	B2 (C1)	3 (75.0) - Undertreated
		2	B2 (C1)	C1 (C2)	
		1	B2 (C1)	B2 (C1)	1 (25.0) - Concordance

Supplementary Table 3.1. Type of hysterectomy (simple vs radical) suggested (conization + MRI VS resurgery specimen) according SHAPE trial

Total no, of patients: 99	**No of patients (%)**	**overtreated / undertreated/ concordance**
**Invasive and minimally invasive** as positive	**3 (3.3)**	**OVERTREATED**
	**6 (6.1)**	**UNDERTREATED**
	**90 (90.9)**	**CONCORDANCE**
**Invasive** as positive (minimally invasive as negative)	**6 (6.1)**	**OVERTREATED**
	**3 (3.0)**	**UNDERTREATED**
	**90 (90.9)**	**CONCORDANCE**

Supplementary Table 3.2 Type of hysterectomy (simple vs radical) suggested (conization + MRI VS resurgery specimen) according SHAPE trial.

Total no. of patients: 99		**No of patients**	**Type of hysterectomy based on tumor size at conization + MRI**	**Type of hysterectomy based on tumor size at conization + resurgery**	**No. of patients (%) - overtreated / undertreated/ concordance**
**TRUE POSITIVE**	**True positive (34)** *considering invasive and minimally invasive as positve*	3	RADICAL	SIMPLE	3 (8.8) – Overtreated
		3	SIMPLE	RADICAL	3 (8.8) – Undertreated
		14	SIMPLE	SIMPLE	28 (82.4) - Concordance
		14	RADICAL	RADICAL	
	**True positive (19)** *considering only invasive as positive (considering minimally invasive as negative)*	2	RADICAL	SIMPLE	2 (10.5) – Overtreated
		2	SIMPLE	RADICAL	2 (10.5) – Undertreated
		4	SIMPLE	SIMPLE	15 (79.0) - Concordance
		11	RADICAL	RADICAL	
**TRUE NEGATIVE**	**True negative (46)** *considering invasive and minimally invasive as positive*	46	SIMPLE	SIMPLE	46 (100.0) – Concordance
	**True negative (57)** *considering only invasive as positive (considering minimally invasive as negative)*	57	SIMPLE	SIMPLE	57 (100.0) – Concordance
**FALSE POSITIVE**	**False positive (4)** *considering invasive and minimally invasive as positive*	4	SIMPLE	SIMPLE	4 (100.0) – Concordance
	**False positive (19)** *considering only invasive as positive (considering minimally invasive as negative)*	4	RADICAL	SIMPLE	4 (21.1) - Overtreated
		15	SIMPLE	SIMPLE	15 (78.9) - Concordance
**FALSE NEGATIVE**	**False negative (15)** ***considering invasive and minimally invasive as positive***	3	SIMPLE	RADICAL	3 (20.0) – Undertreated
		11	SIMPLE	SIMPLE	12 (80.0) - Concordance
		1	RADICAL	RADICAL	
	**False negative (4)** *considering only invasive as positive (considering minimally invasive as negative)*	1	SIMPLE	RADICAL	1 (25.0) - Undertreated
		1	RADICAL	RADICAL	3 (75.0) - Concordance
		2	SIMPLE	SIMPLE	

STARD 2015 Checklist

Section & Topic	No	Item	Reported on page #
**TITLE OR ABSTRACT**			
	**1**	Identification as a study of diagnostic accuracy using at least one measure of accuracy (such as sensitivity, specificity, predictive values, or AUC)	1
**ABSTRACT**			
	**2**	Structured summary of study design, methods, results, and conclusions (for specific guidance, see STARD for Abstracts)	1
**INTRODUCTION**			
	**3**	Scientific and clinical background, including the intended use and clinical role of the index test	2
	**4**	Study objectives and hypotheses	2
**METHODS**			
Study design	**5**	Whether data collection was planned before the index test and reference standard were performed (prospective study) or after (retrospective study)	3
Participants	**6**	Eligibility criteria	3
	**7**	On what basis potentially eligible participants were identified (such as symptoms, results from previous tests, inclusion in registry)	3
	**8**	Where and when potentially eligible participants were identified (setting, location and dates)	3
	**9**	Whether participants formed a consecutive, random or convenience series	3
Test methods	**10a**	Index test, in sufficient detail to allow replication	3
	**10 b**	Reference standard, in sufficient detail to allow replication	3
	**11**	Rationale for choosing the reference standard (if alternatives exist)	n/a
	**12a**	Definition of and rationale for test positivity cut-offs or result categories of the index test, distinguishing pre-specified from exploratory	n/a
	**12 b**	Definition of and rationale for test positivity cut-offs or result categories of the reference standard, distinguishing pre-specified from exploratory	n/a
	**13a**	Whether clinical information and reference standard results were available to the performers/readers of the index test	3
	**13 b**	Whether clinical information and index test results were available to the assessors of the reference standard	3
Analysis	**14**	Methods for estimating or comparing measures of diagnostic accuracy	3
	**15**	How indeterminate index test or reference standard results were handled	n/a
	**16**	How missing data on the index test and reference standard were handled	n/a

	**17**	Any analyses of variability in diagnostic accuracy, distinguishing pre-specified from exploratory	4
	**18**	Intended sample size and how it was determined	3
**RESULTS**			
Participants	**19**	Flow of participants, using a diagram	Figure 3
	**20**	Baseline demographic and clinical characteristics of participants	6 and table 1
	**21a**	Distribution of severity of disease in those with the target condition	6 and table 1
	**21 b**	Distribution of alternative diagnoses in those without the target condition	n/a
	**22**	Time interval and any clinical interventions between index test and reference standard	6 and table 1
Test results	**23**	Cross tabulation of the index test results (or their distribution) by the results of the reference standard	Table 3
	**24**	Estimates of diagnostic accuracy and their precision (such as 95% confidence intervals)	Table 3
	**25**	Any adverse events from performing the index test or the reference standard	n/a
**DISCUSSION**			
	**26**	Study limitations, including sources of potential bias, statistical uncertainty, and generalisability	8
	**27**	Implications for practice, including the intended use and clinical role of the index test	8-9
**OTHER INFORMATION**			
	**28**	Registration number and name of registry	n/a
	**29**	Where the full study protocol can be accessed	n/a
	**30**	Sources of funding and other support; role of funders	Disclosure-Paragraph
